# Characterization of Current Utilization of Machine Perfusion and Living-Donor Liver Transplantation for Hepatocellular Carcinoma

**DOI:** 10.7759/cureus.98365

**Published:** 2025-12-03

**Authors:** Elana Taute, Nina L Eng, Sean P Martin

**Affiliations:** 1 Department of Surgery, Penn State Health Milton S. Hershey Medical Center, Hershey, USA

**Keywords:** hepatocellular carcinome, liver transplantation, living donor liver transplantation, machine perfusion, transplant waitlist

## Abstract

Background: Patients diagnosed with hepatocellular carcinoma (HCC) have experienced a decreased rate of deceased-donor transplants. We aimed to examine differences between two alternative approaches: living-donor liver transplantation (LDLT) and machine perfusion (MP).

Methodology: Using national data, we identified 935 patients who underwent liver transplantation for a primary diagnosis of HCC between January 1, 2017, and December 31, 2023, using either MP or LDLT.

Results: A total of 537 patients were transplanted using MP compared with 398 with LDLT. Throughout the study period, patients transplanted using MP were more likely to be older (odds ratio (OR) 1.03, *P* < 0.01), obese (OR 1.53, *P* = 0.02), and diabetic (OR 1.62, *P* = 0.01) compared with those who received LDLT. Additionally, those receiving MP were less likely to have moderate (OR 0.1, *P *< 0.01) or good (OR 0.1, *P* < 0.01) function status. Despite a worse preoperative condition, patients with MP had a significantly shorter length of stay than those who underwent LDLT (mean 9.6 days vs. 15.8 days, *P *< 0.01) with comparable graft failure rates (MP 3.1% vs. LDLT 3.6%, *P *= 0.7). Additionally, in those patients with available follow-up data, rehospitalization rates were similar between the two groups (MP 43.8% vs. LDLT 43.3%, *P *= 0.89).

Conclusions: Increased competition for organs after the implementation of acuity circle allocation has resulted in decreased rates of transplantation for HCC. LDLT has been one solution to increase access and remains a viable option for some patients. Conversely, MP may help centers offer organs to patients who are non-ideal living donor candidates without concern for worse outcomes.

## Introduction

Hepatocellular carcinoma (HCC) is estimated to be the primary cause in more than 80% of primary liver cancers worldwide [1]. For patients with HCC within Milan criteria, liver transplantation is the most definitive option as it provides treatment for both their malignancy and underlying liver disease. Recent changes in organ allocation have resulted in decreased transplants for patients with HCC [2]. Patients with HCC now have lower top 5 and top 10 offer rates and higher donation after circulatory death (DCD) offer rates [2]. Given that the demand for organs far outweighs the supply, and priority has shifted away from patients with HCC, transplant centers have needed to shift focus to expanding the donor pool. Two such strategies are through the utilization of an organ with perceived lower quality, and the other is through the utilization of living-donor liver transplantation (LDLT). 

Traditional methods of static cold storage (SCS) for liver preservation from deceased donors have failed to meet the current demand, as this method results in ischemic graft injury and does not allow clinicians the ability to assess graft function before implantation [3]. As a result, DCD organs, as well as donors for non-ideal donors, have traditionally been discarded for fear of complications. One alternative to SCS is through the utilization of machine perfusion (MP). The recent PROTECT trial, utilizing normothermic machine perfusion of deceased livers, demonstrated that this technique was superior to SCS in reducing early allograft dysfunction and ischemic biliary complications, and noted that MP increased the utility of DCD livers [4]. LDLT is an additional option for increasing the organ pool for liver transplant. LDLT has been shown to have comparable rates of biliary complications and graft failures compared to SCS [5]. LDLT plays a major role in the treatment of patients with HCC, with a recent study of over 350 patients with HCC demonstrating excellent five-year survival with LDLT [6].

Both LDLT and MP offer a potential alternative method to transplant a patient with HCC. We set out to examine characteristics of the patient population that underwent liver transplant utilizing LDLT and MP. Additionally, we set out to examine the early postoperative outcomes of these patients. Finally, we explored the trends of the use of these methods since the early clinical trials of MP. We hypothesize that MP use is being adopted by transplant centers, given its ease of use and ability to get patients with HCC lifesaving organs. We also hypothesize that MP allows transplant centers to transplant patients who are not ideal LDLT candidates due to medical comorbidities.

This work was presented at the 2024 American Transplant Congress in Philadelphia, Pennsylvania. 

## Materials and methods

Methodology

The Organ Procurement and Transplantation Network (OPTN) liver data were queried from January 1, 2017, to December 31, 2023, for this retrospective cohort study. This dataset captures all patients in the United States who are listed for transplant, as well as those who have undergone transplant surgery**.** For this study, only patients 18 years of age and older were selected. This time interval was chosen to capture patients utilizing MP from both early clinical trials as well as up to the current FDA approval. Patients with HCC were identified by a primary or secondary diagnosis of HCC as the reason for transplantation. The Liver Data set was then merged with the Deceased Donor Data set. MP patients were identified utilizing the unique variable, which is entered at the time of procurement by the local organ procurement organization. Patients listed for simultaneous liver/kidney transplant were included, while those listed for all other multiorgan transplants (heart/liver, lung/liver, and multivisceral abdominal) were excluded. This methodology was chosen to better compare post-transplant outcomes by excluding traditionally high-risk combined thoracic and abdominal transplants. Patients were also excluded if they were undergoing a re-transplant to better compare transplant outcomes. Demographic, listing characteristics, and postoperative outcomes were collected from unique OPTN variables. Function status as defined by OPTN ranges from 10% to 100 %. For this study, poor functional status was defined as 10%-40%; these patients are all classified as unable to care for self. Moderate functional status was 50-70%; these patients are classified as needing some degree of assistance, and good functional status was 80-100% as these patients can care for themselves. 

Descriptive statistics were calculated for all variables of interest. Continuous variables were summarized using means, whereas categorical variables were summarized using frequency and percentages. Comparisons of categorical variables were performed using the chi-square or Fisher’s exact test, whereas continuous variables were compared with the two-sided Student’s t-test. Multivariable logistic regression modeling was performed using variables with a *P*-value ≤ 0.05. Statistical analyses were performed using STATA 16.1. Variables with *unknown* collection were excluded from that analysis, but the patient as a whole was not excluded from this study.

The data reported here have been supplied by the United Network for Organ Sharing as the contractor for the OPTN. The interpretation and reporting of these data are the responsibility of the author(s) and in no way should be seen as an official policy of or interpretation by the OPTN or the U.S. Government.

## Results

Pretransplant patient characteristics 

A total of 935 patients were identified during the study period. Of these, 537 (57%) were transplanted utilizing MP, while 398 (43%) were transplanted utilizing LDLT (Table 1). From this analysis, female patients (odds ratio (OR) 0.40, *P *< 0.01) as well as patients with moderate (OR 0.10, *P* < 0.01) or good (OR 0.10, *P* < 0.01) function status at the time of transplant were associated with lower MP transplants (Table 2). Conversely, increasing patient age (OR 1.03, *P* < 0.01), body mass index (BMI) over 30 (OR 1.53, *P* = 0.02), diabetes at the time of transplant (OR 1.62, *P* = 0.01), and Black ethnicity (OR 4.51, *P* < 0.01) were associated with the utilization of MP. Importantly, on univariable analysis, no difference was noted in laboratory Model for End-Stage Liver Disease (MELD) scores, with an average of 13.5 in the MP cohort compared with 13.7 in the LDLT cohort (*P* = 0.67) (Table 2).

**Table 1:** Pretransplant patient characteristics. MELD, model for end-stage liver disease; OPTN, organ procurement and transplantation network; TIPS, transjugular intrahepatic portosystemic shunt

Variable		MP (*n* = 537) (57%)	LDLT (*n* = 398) (43%)	*P*-value
Sex				<0.01
	Male	419 (78.0)	260 (65.3)	
	Female	118 (22.0)	138 (34.7)	
Blood group				0.83
	O	259 (48%)	181 (46%)	
	A	203 (38%)	160 (40%)	
	B	58 (11%)	45 (11%)	
	AB	17 (3%)	11 (3%)	
Age (mean, 95% CI)		62.7 (62.1-63.4)	59.6 (58.5-60.8)	<0.01
Weight				<0.01
	BMI <=30	306 (57.0)	269 (67.6)	
	BMI >30	231 (43.0)	129 (32.4)	
Education level				0.38
	None	2 (0.4)	0 (0.0)	
	Grade School	46 (8.9)	29 (7.5)	
	High School/GED	201 (38.7)	138 (35.8)	
	Attend College	130 (25.0)	94 (24.4)	
	Associates/Bachelors	93 (17.9)	76 (19.7)	
	Post Graduate	47 (9.1)	48 (12.5)	
Ethnicity				<0.01
	White	344 (64.1)	288 (72.4)	
	Black	29 (5.4)	11 (2.8)	
	Hispanic	116 (21.6)	60 (15.1)	
	Asian	39 (7.3)	37 (9.3)	
	Native American	7 (1.3)	0 (0.0)	
	Pacific Islander	2 (0.4)	1 (0.3)	
	Multiracial	0 (0.0)	1 (0.3)	
Working at the time of transplant				0.64
	No	314 (64.3)	233 (62.8)	
	Yes	174 (35.7)	138 (37.2)	
Diabetes at transplant				<0.01
	No	307 (57.2)	269 (67.6)	
	Yes	230 (42.8)	129 (32.4)	
Encephalopathy at transplant				0.02
	None	339 (63.1)	279 (70.3)	
	Grade 1-2	176 (32.8)	111 (28.0)	
	Grade 3-4	22 (4.1)	7 (1.7)	
Portal vein thrombus				0.44
	No	433 (84.1)	366 (85.9)	
	Yes	82 (15.9)	55 (14.1)	
Previous abdominal surgery				0.18
	No	263 (51.7)	167 (47.0)	
	Yes	246 (48.3)	188 (53.0)	
Previous TIPS				0.33
	No	461 (92.8)	338 (94.4)	
	Yes	36 (7.2)	20 (5.6)	
Ascites at transplant				0.01
	None	270 (50.3)	240 (60.4)	
	Slight	203 (37.8)	124 (31.2)	
	Moderate	64 (11.9)	33 (8.3)	
Functional status				<0.01
	Poor	26 (5.1)	4 (1.0)	
	Moderate	177 (34.4)	100 (25.7)	
	Good	311 (60.5)	285 (73.3)	
Albumin				0.08
	>=3.5	287 (53.4)	190 (47.7)	
	<3.5	250 (46.6)	208 (52.3)	
Creatinine at listing				<0.01
	<= 1	361 (67.2)	300 (75.4)	
	>1	176 (32.8)	98 (24.6)	
MELD at transplant (mean, 95% CI)		13.5 (12.9-14.1)	13.7 (13.1-14.3)	0.67
OPTN region				<0.01
	1	58 (10.8)	30 (7.5)	
	2	16 (3.0)	124 (31.2)	
	3	31 (5.8)	4 (1.0)	
	4	103 (19.2)	59 (14.8)	
	5	172 (32.0)	55 (13.8)	
	6	3 (0.6)	10 (2.5)	
	7	14 (2.6)	19 (4.8)	
	8	46 (8.6)	7 (1.8)	
	9	19 (3.5)	52 (13.1)	
	10	35 (6.5)	18 (4.5)	
	11	40 (7.5)	20 (5.0)	

**Table 2:** Clinical outcomes of recipients of machine perfusion (MP) and living-donor liver transplantation (LDLT).

Variables		MP, *n* (%)	LDLT, *n* (%)	*P*-value
Rejection before discharge				0.17
	No	495 (96.5)	370 (94.6)	
	Yes	18 (3.5)	21 (5.4)	
Rejection within six months				0.01
	No	259 (96.3)	261 (90.9)	
	Yes	10 (3.7)	26 (9.1)	
Rejection within one year				0.06
	No	169 (96.0)	234 (91.4)	
	Yes	7 (4.0)	22 (8.6)	
Graft failure				0.7
	No	496 (96.9)	376 (96.4)	
	Yes	16 (3.1)	14 (3.6)	
Length of stay				<0.01
	<=1 week	263 (49.0)	136 (34.2)	
	>1 week	274 (51.0)	262 (65.8)	
Length of stay (mean, 95% CI)		9.6 (8.7-10.5)	15.8 (12.5-19.1)	<0.01

Utilization of techniques 

**Figure 1:**
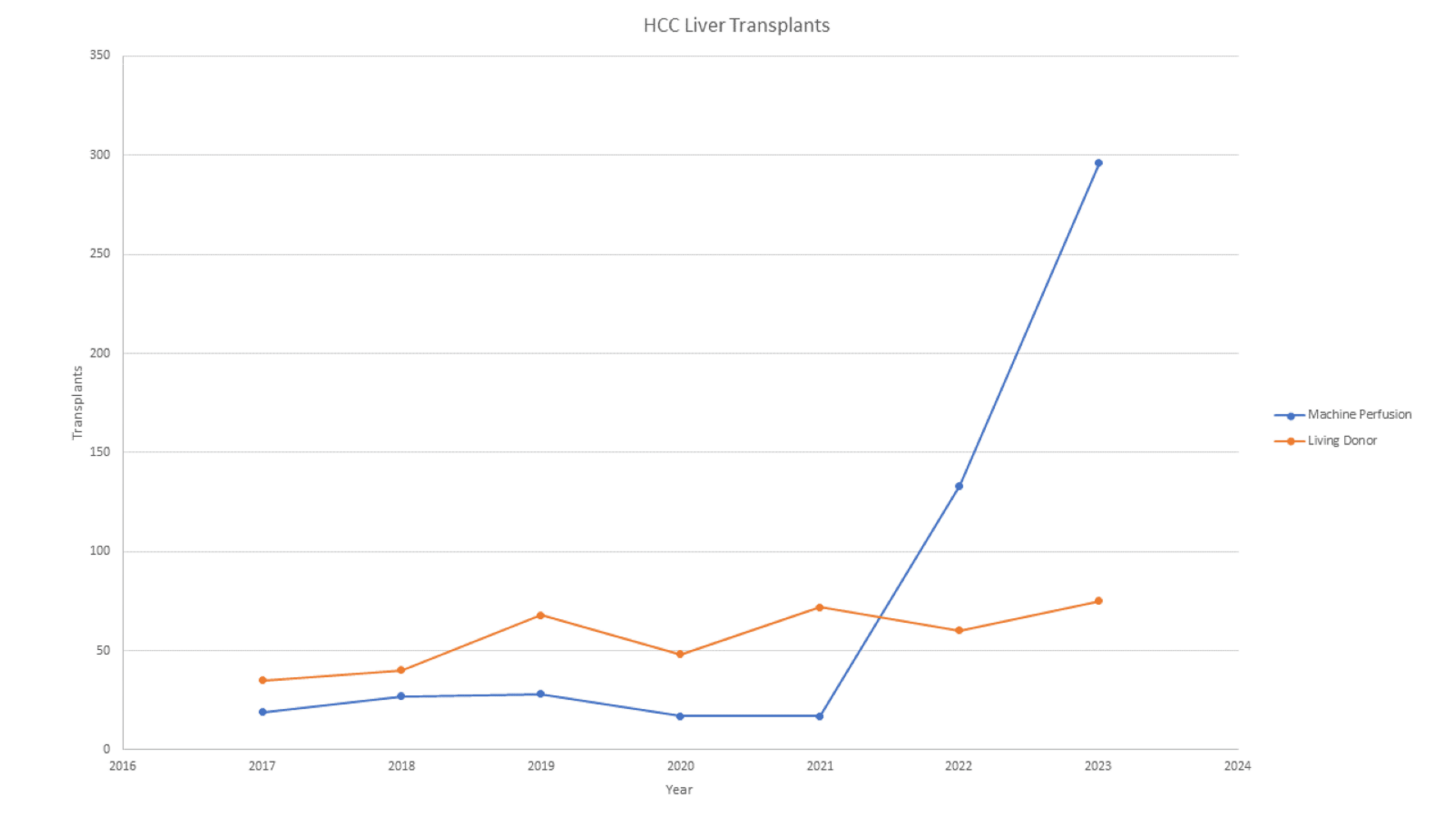
Total number of liver transplants performed by year.

**Figure 2:**
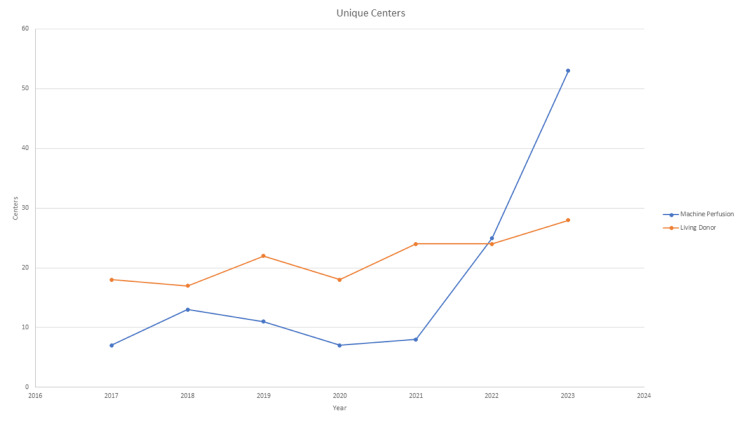
Total number of unique centers.

Over the study period, a 1457.9% increase in MP utilization was observed, coinciding with its transition from an experimental technology to FDA approval (Figure 1). The largest increase occurred from 2021 to 2022, during which cases rose from 17 to 133. Conversely, LDLT cases increased by only 114.3% over the study period, with overall numbers remaining relatively stable during the past three years. With the rising number of MP cases, this technique is now being utilized in 53 unique centers, representing a 657.1% increase overall and a 112% increase from 2022 to 2023. LDLT use increased from 18 centers to 28 centers over the study period, a 55.5% increase, with only four new centers adopting this technique between 2021 and 2023 (Figure 2).

Clinical outcomes 

No difference in rejection episodes before discharge was observed; however, rejection events within six months were significantly higher in the LDLT cohort compared with the MP cohort (9.1% vs 3.7%, *P* = 0.01), although this difference was not seen at one year. Additionally, while rejection events were more common, this did not lead to increased graft failure, which was similar in both groups (LDLT 3.6% vs. MP 3.1%, *P *= 0.7). Overall length of stay for the MP cohort was significantly shorter as compared to the LDLT cohort (9.6 days vs. 15.8 days, *P* < 0.01) (Table 3).

**Table 3:** Odds ratios of recipient characteristics receiving machine perfusion (MP). BMI, body mass index; OPTN, Organ Procurement and Transplantation Network

Variable		Odds ratio	95% CI	*P*-value
Sex				
	Male	ref		
	Female	0.40	0.27-0.60	<0.01
Age		1.03	1.01-1.04	<0.01
BMI				
	<=30	ref		
	>30	1.53	1.06-2.20	0.02
Ethnicity				
	White	ref		
	Black	4.51	1.64-12.38	<0.01
	Hispanic	1.18	0.73-1.89	0.50
	Asian	0.60	0.32-1.15	0.13
	Pacific Islander	0.48	0.03-7.04	0.60
Diabetes at transplant				
	No	ref		
	Yes	1.62	1.12-2.33	0.01
Encephalopathy at transplant				
	No	ref		
	Yes	1.01	0.70-1.47	0.94
Ascites at transplant				
	No	ref		
	Yes	1.28	0.94-1.75	0.11
Functional status at transplant				
	Poor	ref		
	Moderate	0.10	0.03-0.35	<0.01
	Good	0.10	0.03-0.36	<0.01
Elevated creatinine				
	Normal	ref		
	Elevated	1.15	0.78-1.71	0.48
Days on waiting list		1.00	1.00-1.00	<0.01
OPTN region				
	1	ref		
	2	0.09	0.04-0.19	<0.01
	3	5.39	1.57-18.45	0.01
	4	1.15	0.60-2.21	0.67
	5	3.21	1.68-6.14	<0.01
	6	0.30	0.05-1.68	0.17
	7	0.52	0.20-1.36	0.18
	8	8.22	2.98-22.72	<0.01
	9	0.25	0.11-0.57	<0.01
	10	2.17	0.94-5.00	0.07
	11	1.39	0.63-3.06	0.42

## Discussion

Following the national change in donor allocation, liver transplantation for patients with HCC has decreased [2]. Our study aims to characterize the utilization of two alternative approaches to access for donor organs, MP and LDLT. From our data, we observed that nationally, MP was more commonly associated with patients of older age, higher BMI, and with moderate to poor preoperative functional status. We also observed that despite representing a more challenging patient population, these patients had shorter length of stay, with comparable postoperative outcomes. Additionally, we have observed a greater overall trend in the utilization of MP as compared to a much slower overall growth in LDLT for HCC.

Patient selection and donor matching are the first steps in achieving positive outcomes after organ transplantation. The decision to place a patient on the living donor transplant list must be approached with even greater thoughtfulness as the best interest of the living donor must also be considered. A recent American Society of Transplantation consensus conference noted that among some of the greatest challenges to LDLT are the lack of uniform awareness and utilization of LDLT among many subgroups of patients, including those with HCC and those with increased frailty scores [7]. Similarly, a survey study of transplant centers in the United States noted heterogeneity in candidate exclusion criteria for LDLT, with age caps in some centers as low as 65 and BMI cutoffs of as low as 30 [8]. Our findings of utilization trends mirror these studies and suggest that the LDLT is primarily used for younger and non-obese patients. Our data suggest that MP is being utilized for patients who would often be considered non-ideal LDLT candidates. 

Another aspect of patient selection for transplantation is the current state of their overall health, including their degree of frailty. Frailty is associated with a 62% increased risk of mortality following liver transplant, and patients classified as frail had significantly longer lengths of stay and are more than twice as likely to require advanced care in a facility following hospital discharge [9]. Even before undergoing the stress of surgery itself, frailty is associated with increased risk of death while on the transplant waitlist [10]. It is therefore paramount that patients receive a transplant to correct the underlying condition driving the source of their frailty. LDLT has been shown to benefit high-risk waitlisted candidates by allowing for earlier access to organs, yet the practice of utilizing living donors for high-risk patients, including those with increased frailty scores, is not formalized or widely accepted [8,11]. Our data coincide with survey data from transplant centers, indicating that LDLT is being used in patients with the highest level of functional status. From our data, transplant centers are utilizing MP for patients with moderate to poor functional status. This frail population is benefiting from MP technology as a way to get access to organs. Interestingly, when examining postoperative outcomes, this frail population has a significantly shorter length of stay postoperatively despite classically higher surgical risk. These data suggest that for patients deemed too frail for LDLT, MP may be a safe alternative to gain access to transplant.

Social determinants of health also play an important role in access to care for patients with HCC in need of a liver transplant. Multiple studies have demonstrated that patients of ethnic minorities and those patients in geographically isolated regions are less likely to undergo transplantation, as well as more likely to die on the transplant waitlist [12,13]. Specifically focusing on LDLT, African American patients represent a small proportion of total LDLT, which has been attributed to decreased numbers of potential donor inquiries [14]. The burden to advertise and educate communities on the advantages of LDLT falls to transplant centers whose resources are often stretched, resulting in more than 50% of centers providing minimal to no support to patients to help identify donors [8]. Additionally, for centers that do not currently offer LDLT, the high resource burden and surgical expertise required to perform LDLT represent major perceived barriers to adding this modality of donor utilization [8]. From our data, we observed that MP is being utilized in a manner to overcome racial and geographic disparities that have been seen overall in liver transplantation, as well as in LDLT. We noted that African American patients were far more likely to undergo transplant with MP for HCC, as this modality does not require identification of a donor and the associated personal and financial burden associated with donation. In addition, we have noted that this procurement technique is expanded far more rapidly in the modern transplant era, likely in part to the lack of burden placed on the transplant center in regard to staffing and additional surgical expertise. Our data suggest that MP helps to alleviate disparities of previously disadvantaged populations.

While our data provide a national overview of the current state of MP and LDLT, it is limited to those variables collected in the OPTN dataset. Most importantly, our identification of MP cases is limited to those that are captured at the time of procurement and does not consider those cases in which the organ was placed on a machine perfusion device after returning to the transplant institution, a *back-to-base* model. As with all large national datasets, the nuances of the decision to list patients for transplant and organ acceptance cannot be known; however, this dataset does allow for overall trends to be examined.

## Conclusions

The need for life-saving organs continues to outpace availability. The need to continue to expand the donor pool is at the forefront of challenges facing the transplant community. From our data, we have observed that both LDLT and MP are viable options for patients with HCC. Specifically, MP is associated with access to transplantation for patients who may not be ideal LDLT candidates and offers similar early outcomes. These similar outcomes will need to be further investigated in a prospective manner. Additionally, MP may help to alleviate barriers to transplantation for HCC patients from historically disadvantaged populations. As the growth of MP continues, it is important to continue to monitor these trends to ensure that access to care is fair and just.
